# Postural Fitness Protocol in Children and Adolescents: Descriptive Values from the ISQUIOS Program

**DOI:** 10.3390/children12091253

**Published:** 2025-09-17

**Authors:** María Teresa Martínez-Romero, Mark De Ste Croix, Pilar Sainz de Baranda

**Affiliations:** 1 Department of Physical Activity and Sport, Faculty of Sport Sciences, Campus of Excellence Mare Nostrum, University of Murcia, 30720 Murcia, Spain; 2 Sports and Musculoskeletal System Research Group (RAQUIS), University of Murcia, 30100 Murcia, Spain; 3 School of Education, Sport and Applied Sciences, University of Gloucestershire, Gloucester GL2 9HW, UK; mdestecroix@glos.ac.uk

**Keywords:** students, posture, spine, hip joint, trunk muscles

## Abstract

**Highlights:**

**What are the main findings?**

- There are clear sex-related differences in spinal morphology, hip flexibility, and trunk muscle function.
- These results emphasize the need to consider sex as a determining factor when assessing posture and designing physical screening protocols for young people.

**What are the implications of the main findings?**

- The “Postural Fitness” protocol not only may serve as a valuable and feasible tool for early screening of postural deviations and physical deficits but also offers a foundation for individualized preventive strategies.
- Educators and practitioners should incorporate spine health education, functional mobility work, and muscular endurance challenges into youth physical activity programs, with special attention to pubertal changes and postural evolution.

**Abstract:**

Background/Objectives: Back pain is increasingly prevalent during childhood and adolescence, often predicting adult spinal disorders. This study aimed to describe sex-specific anthropometric and “Postural Fitness” characteristics in school-aged children and adolescents and to introduce a standardized, field-based assessment protocol for early screening of postural and functional deficits. Methods: This cross-sectional study included a total of 494 students (8–17 years; 50% girls) from 14 schools in Murcia (Spain). Exclusion criteria included diagnosed spinal pathology or major physical injury, lack of signed informed consent, absence on the testing day, and incomplete Postural Fitness assessment. The “Postural Fitness” protocol included assessments of sagittal spinal alignment (inclinometer), hip range of motion (ROM) (inclinometer with an extendable telescopic arm), pelvic tilt (goniometer with a spirit level system), and trunk muscle endurance (chronometer). Tests were conducted in physical education sessions by trained sports scientists. Results: Significant sex-based differences were observed. Boys exhibited greater thoracic kyphosis (40.3 ± 9.6° vs. 36.7 ± 9.2°), reduced hip ROM (passive hip extension (PHE): 16.8 ± 8.1°, passive hip flexion with knee extension (PHFKE): 68.9 ± 8.6°), and more posterior pelvic tilt (104.9 ± 8.4° vs. 99.7 ± 8.1°), whereas girls demonstrated increased lumbar lordosis (35.7 ± 8.6° vs. 31.5 ± 8.5°), greater hip ROM (PHE: 18.5 ± 9°, PHFKE: 77.9 ± 13°), and superior trunk extensor endurance (123.2 ± 74.7 s vs. 106.2 ± 69.8 s). Lateral trunk muscle endurance was higher in boys (48.7 ± 31 s vs. 41.4 ± 24.9 s). Conclusions: The “Postural Fitness” protocol proved feasible in school settings and revealed key sex-based disparities in spinal and neuromuscular profiles. These findings highlight the need for individualized, sex-specific screening and preventive programs to enhance back health during growth. Implementing this protocol may support early identification of modifiable risk factors linked to spinal dysfunction and pain in youth.

## 1. Introduction

Back pain (BP) is a very frequent reason for consultation among adults, but it is also increasingly common in children and adolescents. Epidemiological studies report that lifetime prevalence rises from around 1% at age 7 to 12–40% by age 12, increasing sharply during adolescence (39–71% between 12 and 15 years), coinciding with pubertal changes [1]. By the end of adolescence, BP prevalence approaches that observed in adults [1,2]. Longitudinal evidence further shows that the presence of BP during adolescence is a strong predictor of BP in adulthood [3,4,5], underscoring the importance of early identification of risk factors [6,7,8].

The etiology of BP is multifactorial, involving biological, psychological, biomechanical, and behavioral determinants [6,7,8]. Some factors, such as sex, age, pubertal status, family history, and height, are non-modifiable [9]. However, a growing body of evidence highlights modifiable factors—including body composition, posture, flexibility, trunk muscle endurance, physical activity, sedentary behavior, and sleep quality—as key targets for prevention [2,9,10,11].

Within these modifiable determinants, physical and postural variables have gained increasing attention. Limited flexibility, especially in the hamstrings, iliopsoas, and quadriceps, has been consistently associated with BP in youth [2,11,12]. For example, Endo et al. [12] reported that elementary school softball players with quadriceps tightness had significantly higher odds of experiencing BP. Similarly, reduced finger-to-floor distance has been linked to BP in children aged 6–12 [2]. Trunk muscle endurance, sagittal spinal alignment, spinal mobility, and neurodynamic assessments have also been identified as correlates of adolescent BP [6,7]. Decreased trunk muscular endurance—both in flexor and extensor muscles—has been associated with the presence of low back pain (LBP) [7,13,14]. Likewise, sagittal spinal misalignments, such as increased thoracic kyphosis, a more pronounced lumbar lordotic apex, or greater pelvic retroversion, have been reported as risk factors for LBP in adolescents [7,15]. In addition, excessive sedentary behavior, poor lifting techniques, and asymmetrical backpack use increase susceptibility to BP [3,16].

Currently, several test batteries are available to assess health-related physical fitness in children and adolescents. These batteries primarily enable physical education (PE) teachers to evaluate cardiovascular fitness and muscular strength [17,18,19]. However, postural health and back care are most often assessed through questionnaires, which may overlook key physical determinants. To address this gap, our research group has introduced the concept of “Postural Fitness”, an integrative construct that reflects the ability of children and adolescents to maintain musculoskeletal health through adequate spinal alignment, flexibility, mobility, and trunk muscle endurance. Based on this concept, the “Postural Fitness” protocol was developed, a comprehensive test battery that combines validated and reliable physical tests to assess the main modifiable factors associated with postural health identified in the scientific literature [2,6,7,14,15]. This initiative aims to facilitate early detection of spinal disorders and to support the prevention of back pain in school-aged populations.

Therefore, the aims of the present study were twofold: (a) to describe anthropometric characteristics and “Postural Fitness” variables—including sagittal spinal alignment, spinal mobility, hip range of motion (ROM), and trunk muscle endurance—in a sample of school-aged children and adolescents, stratified by sex; and (b) to present and operationalize the “Postural Fitness” protocol as a standardized screening tool. The hypotheses of the present study were (a) females present a hyperlordotic lumbar morphotype and higher flexibility than boys, and (b) boys present a hyperkyphotic thoracic morphotype and higher trunk muscle endurance than girls.

## 2. Materials and Methods

### 2.1. Design

**Figure 1:**
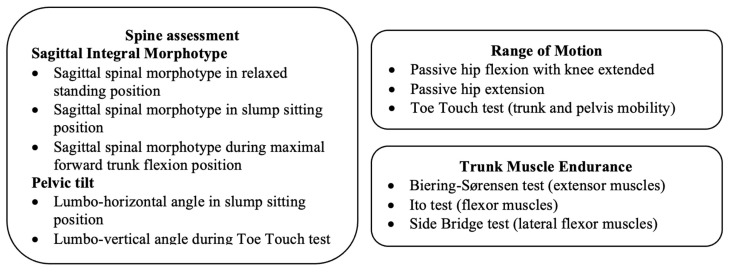
Physical variables included in the “Postural Fitness” protocol.

This cross-sectional study involved the collection of all measurements from the “Postural Fitness” protocol (Figure 1) prior to participants’ enrolment in the postural and physical fitness intervention known as the “ISQUIOS Program”. The study design, protocol, and methodology were approved by the Ethics Review Committee for Research Involving Human Subjects at the University of Murcia (Spain) (ID: 1920/2018), in accordance with the principles outlined in the Declaration of Helsinki (1961), revised in Fortaleza (2013). Students, parents/guardians, and PE teachers were fully informed verbally and in writing about the nature and purpose of this study. All parents/guardians signed an informed consent form prior to participation.

### 2.2. Participants

**Figure 2:**
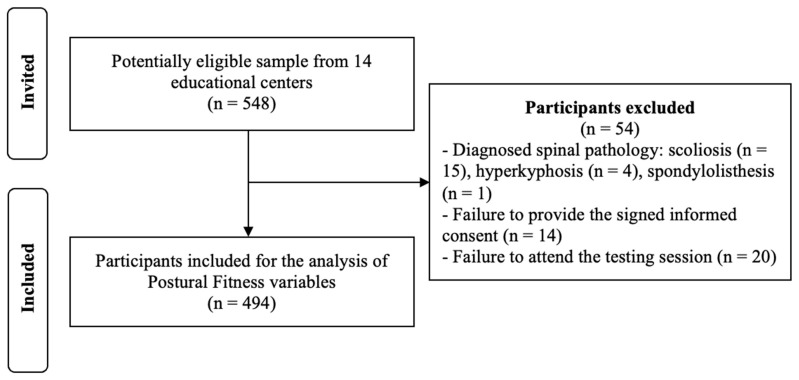
Flow diagram of participant selection.

An a priori power analysis (G*Power 3.1, Franz-Faul, Universität Kiel, Germany) showed that 130 participants per group (males and females) were required to detect an effect size of d = 0.35 with α = 0.05 and power = 0.80 for an independent two-sample *t*-test (two-tailed). The sample was increased by 10% to account for potential dropouts. A total of 548 students aged 8 to 16 years were initially invited to participate in this study through a convenience sampling strategy, involving 14 different educational centers from the Region of Murcia (Spain). The exclusion criteria were as follows: (a) diagnosis of spinal pathology or a significant physical injury that limited the correct execution of the tests; (b) failure to return the signed informed consent form (from both parents/guardians and students) prior to the start of this study; (c) absence on the day of data collection; or (d) incomplete “Postural Fitness” assessment. Based on these exclusion criteria, 54 of the initially invited participants were removed, and a total of 494 students (age: 11.03 ± 1.48 years; range: 9–17 years; 50% female) were finally included for the analyses (Figure 2).

### 2.3. Procedure

**Figure 3:**
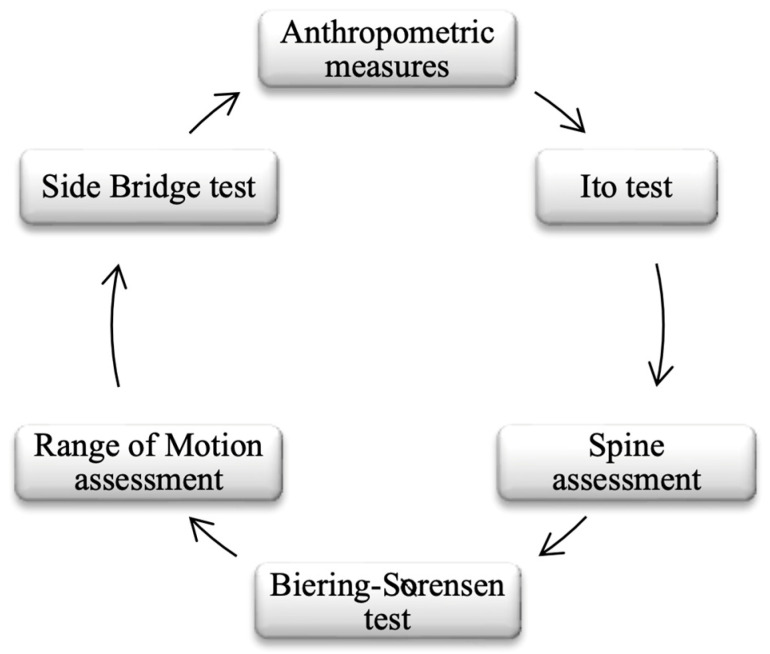
Structure of the assessment circuit conducted during the testing session.

Participants were assessed during one of their regular morning PE lessons. Given that PE teachers have only two 60 min sessions per week for each grade level, an efficient circuit-based setup was implemented during the testing session to carry out the “Postural Fitness” protocol along with anthropometric measurements. Six different stations were arranged for this purpose (Figure 3).

Participants were instructed to refrain from engaging in strenuous physical activity during the 24 h prior to testing. In addition, to reduce the onset of muscular fatigue during trunk muscle endurance testing, the specific stations were strategically alternated with the spine posture assessment, ROM protocol, and anthropometric measurement stations. This organization ensured an average rest period of approximately 5 min between each trunk muscle test.

All testing was conducted in an indoor sports facility under standardized conditions at 25°C. Participants were assessed wearing sports clothing and barefoot, except during the evaluation of spinal curvatures, for which they wore only underwear in an enclosed room. At the beginning of the testing session, all participants received detailed verbal instructions regarding the testing procedures, and any questions were addressed by the research team.

Data collection was performed by a team of seven researchers, all of whom were specialists in Sports Science with over five years of experience in neuromuscular performance assessment.

#### 2.3.1. Anthropometric Measures

Body mass (kg) was measured using a calibrated physician scale (SECA 799, Hamburg, Germany), and standing height (cm) was recorded using the integrated stadiometer of the same device. Sitting height (cm) was also measured using a standard measurement platform. Leg length was calculated as the difference between standing height and sitting height. BMI was computed as body mass divided by height squared (kg/m^2^). All anthropometric measurements were conducted by the same trained rater.

#### 2.3.2. Postural Fitness Protocol

The “Postural Fitness” protocol (Figure 1) is grounded in the evaluation of key physical factors affecting back health, through the assessment of sagittal spinal curvatures and pelvic tilt [20], hip ROM [21], and trunk muscle endurance [22,23,24]. Prior to the testing session, a double-blind study was conducted with 10 participants to establish rater reliability. Two assessment sessions were performed 24 h apart, showing intraclass correlation coefficients ranging from 0.93 to 0.98 for all sagittal spinal and pelvis measurements, and ranging from 0.95 to 0.98 for ROM measurements. In relation to trunk endurance tests, PE teachers attended a practical workshop to learn and standardize the trunk tests and subsequently practiced them with students at least four times before data collection. This was based on previous studies in high school students [25], which highlighted acceptable reliability (ICC > 0.75) but learning effects when fewer trials were performed. Thus, by the assessment day, participants were familiar with the tests.

1. Sagittal Integral Morphotype (SIM)

**Figure 4:**
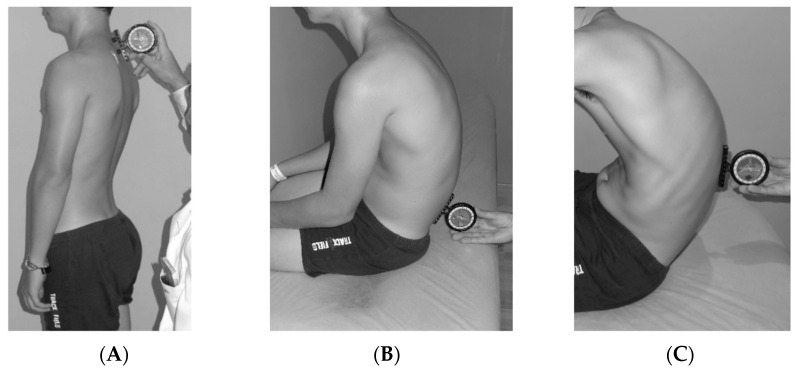
SIM assessment: (**A**) relaxed standing position (SP); (**B**) slump sitting position (SSP); (**C**) maximal forward trunk flexion position (FTFP).

Sagittal spinal curvatures (thoracic and lumbar regions) were assessed using the SIM protocol, as described by Santonja-Medina et al. [20], which provides a comprehensive evaluation of sagittal spinal alignment (Figure 4). This assessment encompasses three standardized postural positions: relaxed standing position (SP), slump sitting position (SSP), and maximal forward trunk flexion position (FTFP). The protocol was specifically developed to facilitate an accurate and thorough diagnosis of sagittal spinal alignment deviations [26,27]. A comprehensive description of the procedure is available in Santonja-Medina et al. [20].

An inclinometer (ISOMED Inc., Portland, OR, USA) was employed to quantify sagittal spinal curvatures. This instrument has demonstrated high reproducibility and validity, showing good correlation with radiographic measurements [28].

Reference values for each curvature and position are presented in Table 1, where negative values represent degrees of posterior concavity (lordosis), and positive values indicate anterior concavity (kyphosis) [20].

Table 2 and Table 3 present the classification and subclassification criteria for the comprehensive sagittal diagnosis of the thoracic and lumbar spine, respectively. This diagnosis is determined based on the values obtained from the three measured positions [20].

2. Pelvic tilt and Toe Touch test

**Figure 5:**
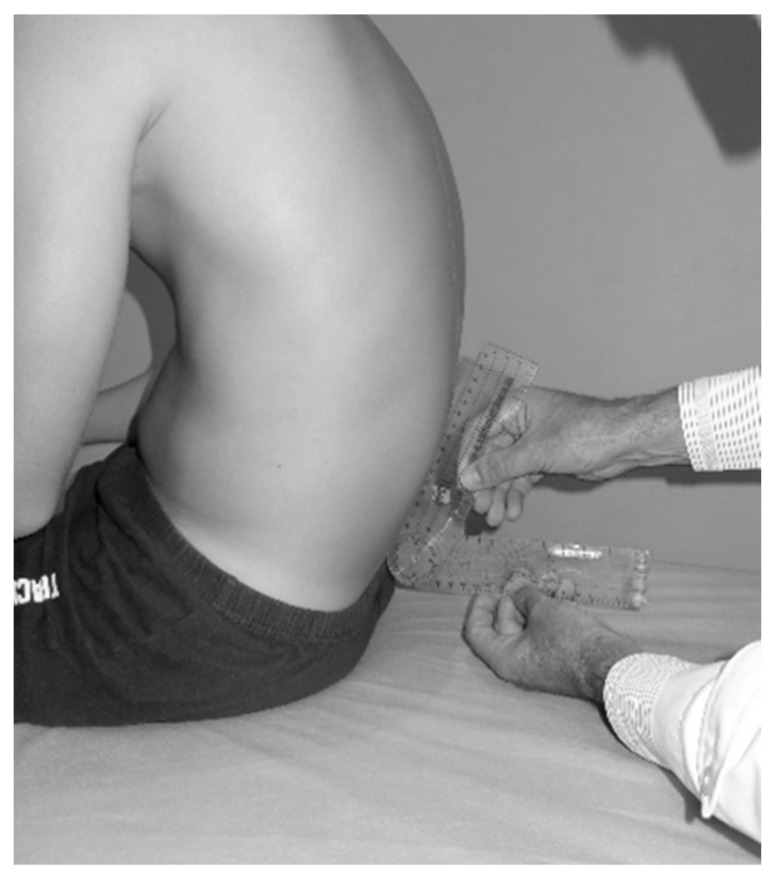
Pelvic tilt assessment through L-H angle.

Pelvic tilt was assessed using the lumbosacral angle in both the SSP (Figure 5) and during the TT test. This assessment allowed for determining whether students were able to maintain pelvic verticality—and consequently a more neutral sagittal spine—while sitting in an SSP or bending the trunk forward. The lumbosacral angle was measured using a goniometer equipped with a spirit level system (GonioSant©, Bubble Level Plastic Goniometer 180 DEG 7.5, Imucot Traumatología S.L., Murcia, Spain), following the method validated and described by Ayala et al. [29] and Sainz de Baranda et al. [30]. The angle was formed between either a horizontal (lumbo-horizontal angle, L-H) or vertical (lumbo-vertical angle, L-V) reference line and the alignment of the spinous processes from L4 to S1. For data analysis, the supplementary angle was used.

Pelvic tilt was classified as normal when the angle was ≤100°, and as posterior pelvic tilt when the angle was >101°, in line with previous classifications [29,31]. All assessments of the spine and pelvic tilt were performed by the same rater at the same measurement station.

TT test: This test was used to evaluate the mobility of the entire spine and pelvis during forward trunk flexion, as well as to indirectly assess hamstring flexibility [32,33]. Participants stood barefoot with their feet hip-width apart on the Sit and Reach box and were instructed to bend forward as far as possible while keeping the knees, arms, and fingers fully extended over the measuring scale. The vertical distance reached by the fingertips was recorded in centimeters. This test complements the L-V angle assessment by providing additional information on the relative contribution of spinal and pelvic mobility during forward trunk flexion [29]. The test was conducted at the spinal assessment station by the same rater to ensure consistency.

3. Range of motion

Two tests from the ROM-SPORT battery were used to evaluate hip joint ROM, following the protocol validated and described by Cejudo [21,34]. Measurements were taken using an inclinometer (ISOMED Inc., Portland, OR, USA) equipped with an extendable telescopic arm. To ensure pelvic neutrality during testing, a lumbar support (Lumbosant©, Murcia, Spain) was used in all tests. Each ROM test was performed twice on both limbs, and the mean value of the two trials was used for statistical analysis [21]. All evaluations were conducted at the same station by two trained raters (a principal and an assistant). The endpoint of each test was determined by one or more of the following criteria: (a) the principal rater could not continue the passive movement due to increasing resistance from the target musculature; (b) compensatory movements were observed by one or both raters; (c) the participant reported a strong but tolerable stretching sensation, just before the onset of pain [21].

**Figure 6:**
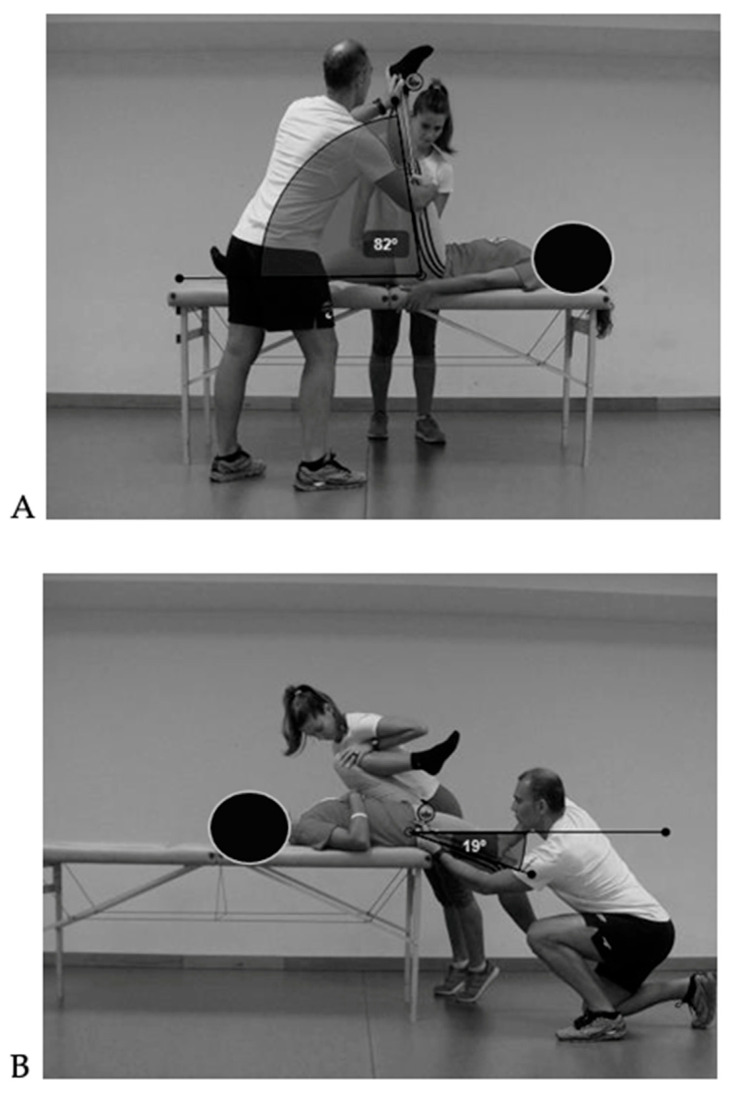
(**A**) Hip extensor muscles assessment through the PHFKE; (**B**) hip flexor muscles assessment through the PHE.

The two selected ROM tests were Passive Hip Flexion with Knee Extended (PHFKE), also known as the Passive Straight Leg Raise test, and Passive Hip Extension (PHE) or Modified Thomas test (Figure 6). A comprehensive description of the procedure is available in Cejudo [21]. PHFKE was classified as normal when the angle was ≥75°, and as reduced when the angle was <75° [35,36]. PHE was classified as normal when the angle was ≥14°, and as reduced when the angle was <13° [36].

4. Trunk muscle endurance

**Figure 7:**
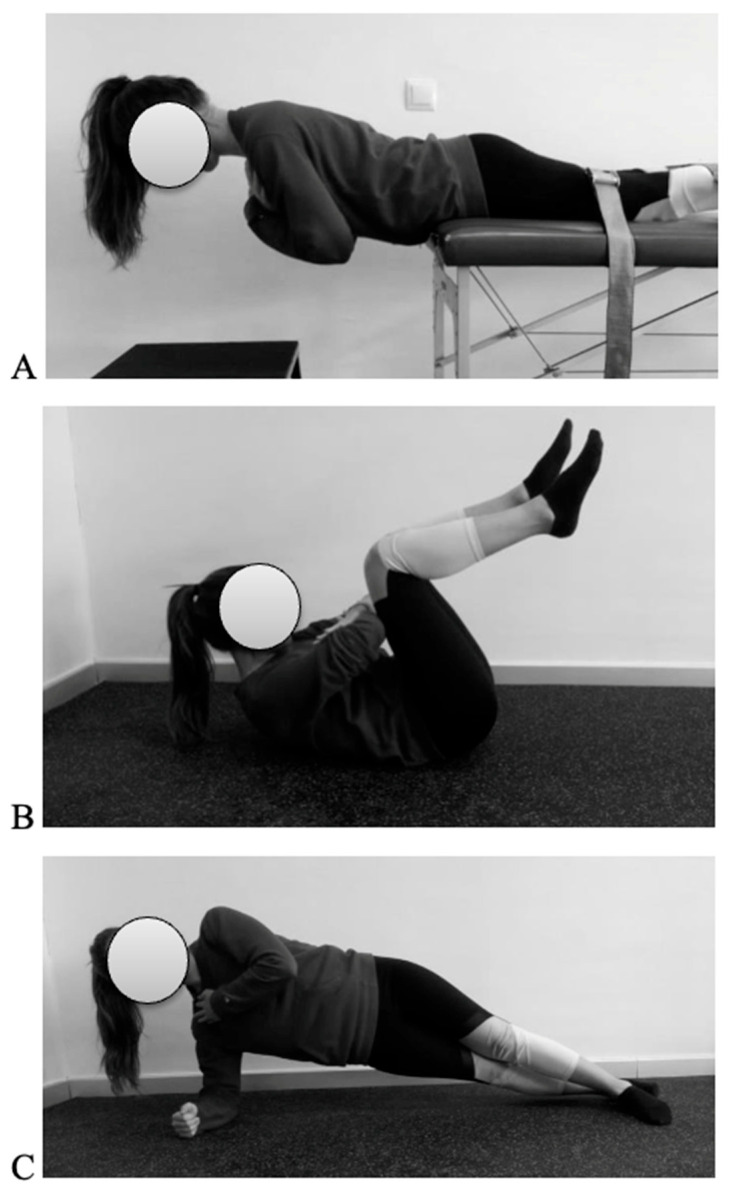
Trunk endurance field-based tests: (**A**) BS test; (**B**) Ito test; (**C**) SB test.

Three validated field-based tests (Figure 7) were selected to assess the isometric endurance of the trunk extensor, flexor, and lateral flexor muscles: the Biering–Sørensen (BS) test [24], the Ito test [22], and the Side Bridge (SB) test [23], respectively. These tests were chosen to evaluate the endurance capacity of different trunk muscle groups and to provide a more comprehensive understanding of core function [25,37]. During each test, participants received strong verbal encouragement to maintain the required position for as long as possible. The duration (in seconds) was recorded as the outcome variable. A comprehensive description of the procedure is available in Martínez-Romero et al. [25,37].

### 2.4. Statistical Analyses

The normality of the distribution for each variable was assessed using the Kolmogorov–Smirnov test. Descriptive statistics were calculated for the total sample and stratified by sex. The results for quantitative variables are presented as means and standard deviations (SD), while qualitative variables are reported as absolute frequencies and percentages.

To analyze differences in continuous variables between independent groups (e.g., males vs. females), independent *t*-tests (for two groups) were applied when data met normality assumptions. For variables that did not follow a normal distribution, the Mann–Whitney U test was used, as this non-parametric test does not require the assumption of normality and is more robust when dealing with skewed distributions, small sample sizes, or ordinal data. Effect size (ES) was calculated as Cohen’s d and interpreted according to the classification proposed by Hopkins et al. [38]: trivial (<0.2), small (0.2–0.59), moderate (0.6–1.19), large (1.20–2.00), very large (2.01–3.99), and extremely large (>4.0).

To control for potential confounding variables, hierarchical linear regression models were performed. Sex (male/female) was first entered into the model as the main independent variable. In a second step, age and body mass index (BMI) were added as covariates in order to adjust for their possible influence on Postural Fitness variables.

Chi-square tests (bivariate analysis) were applied to explore associations between sex (dependent variable) and the qualitative variables. Furthermore, “Phi” and “V of Cramer” statistics were calculated to determine the ES, according to the classification proposed by Kim [39]: small (<0.20), moderate (0.21–0.34), and large (>0.35). For all tests, the significance level was set at *p* < 0.05.

All statistical analyses were performed using SPSS software, version 21.0 for Windows (IBM Corp., Armonk, NY, USA).

## 3. Results

Descriptive statistics for the overall sample and by sex are presented in Table 4. In the “Fitness Postural” assessment, boys presented greater thoracic kyphosis across all evaluated positions. In contrast, girls exhibited greater lumbar lordosis in the SP and less lumbar kyphosis, along with a more neutral pelvic position in both the SSP and forward bending tests, which may reflect sex-related differences in spinal alignment development. In terms of ROM, girls consistently showed a greater range in all tests performed, which may be related to the different alignment of the spine. Finally, in the trunk muscle endurance tests, boys demonstrated higher endurance in the lateral flexor muscles, whereas girls performed better in the trunk extensor test. These patterns highlight potential differences in functional core stabilization strategies between sexes. However, the ES of significant differences was small, ranging from 0.2 to 0.49. After adjusting for age and BMI—factors known to influence spinal curvatures, lower limb ROM, and trunk muscle endurance—the effect of sex on the dependent variables remained statistically significant.

As shown in Table 5, most students exhibited normal thoracic kyphosis in both the SP (57.1%) and SSP (70.6%). However, in the maximal FTFP, the majority of participants (72.2%) presented thoracic hyperkyphosis. When analyzing the data by sex, a similar distribution pattern was observed, although hyperkyphosis in SP and SSP was more prevalent among boys, whereas normal thoracic curvature was more frequent in girls. The classification of the SIM for thoracic curvature revealed that the most prevalent morphotype was “dynamic functional hyperkyphosis”, followed by the “normal” morphotype and “total hyperkyphosis”. An association between “total hyperkyphosis” and male sex was observed, while the “normal” morphotype was more common among females, reinforcing the sex-specific postural tendencies noted above and the distinct compensatory patterns of spinal alignment during static and dynamic positions. However, the ES of significant associations was small, ranging from 0.12 to 0.21.

Regarding lumbar curvature (Table 5), most participants presented a “normal” lumbar profile across all three positions, both in the total sample and when disaggregated by sex. However, hyperlordosis in the SP was more frequently observed in girls, whereas lumbar hyperkyphosis in the SSP was more common in boys. The classification of the SIM for lumbar curvature revealed that the most prevalent morphotype was “normal”, followed by the “hyperlordotic attitude” and “total functional hyperkyphosis”. An association was observed between “hyperlordotic attitude” and females, while “total functional hyperkyphosis” and “functional static hyperkyphosis” were more common among males, also confirming sex-specific postural tendencies for lumbar curvature. However, the ES of significant associations was small to moderate, ranging from 0.15 to 0.22.

In terms of pelvic tilt (Table 6), a posterior pelvic tilt was the predominant pattern, especially in the maximal FTFP. This postural feature, which was significantly more common in boys (ES = 0.25–0.37, moderate), may indicate sex-related differences in lumbopelvic control. Finally, a high proportion of participants showed reduced ROM in both hip flexors and hip extensors, with these limitations being more prevalent in boys for the PHFKE test (ES = 0.28–0.3, moderate). These restrictions may have functional implications on lumbopelvic control and sagittal alignment of the spine.

## 4. Discussion

The primary objective of this study was to examine anthropometric characteristics and key components of the “Postural Fitness” protocol—including sagittal spinal alignment, spinal mobility, hip ROM, and trunk muscle endurance—in children and adolescents, with a focus on sex-based differences. To the best of our knowledge, this is the first study to offer a comprehensive characterization of “Postural Fitness”, including SIM, hip ROM, and trunk muscle endurance in a general population of healthy, non-athletic children and adolescents. This adds relevant baseline information for designing age- and sex-specific screening and preventive interventions.

Spinal evaluations showed that boys exhibited significantly greater thoracic kyphosis across all three assessed positions. Conversely, girls demonstrated more pronounced lumbar lordosis in SP, and a more neutral pelvic tilt, and reduced lumbar kyphosis in SSP and FTFP. These differences align with previously reported sex-based postural patterns, in which girls tend to exhibit more lordotic alignments and boys more hyperkyphotic or sway-back postures [40,41,42,43,44]. Importantly, these deviations are not only structural descriptors but may also reflect early biomechanical tendencies that can influence spinal loading and risk for musculoskeletal symptoms during growth.

In relation to the categorization of the SIM for the dorsal curve, boys presented a higher prevalence of “functional thoracic hyperkyphosis”, whereas the most frequent among girls was “normal”. However, for the lumbar curve, the overall SIM was more frequently “normal” in both sexes, confirming this study’s hypotheses. These findings on dorsal curve are consistent with previous studies using multi-positional assessment [20,45,46]. In relation to lumbar curve, Santonja-Medina et al. [20] reported a much higher percentage of “functional lumbar hyperkyphosis” than observed here (82.3% vs. 33.7%), likely due to the difference in sample size of their study compared to this one (731 vs. 252) and the age range studied (8–12 vs. 8–16).

The literature suggests that both lumbar and thoracic curvatures tend to increase with age [40,42,47]. Therefore, these spinal postural misalignments in children and adolescents, often arising from a combination of incorrect postural habits and imbalances in muscle activity (hypo- or hyperactivity), may compromise both skeletal and soft tissue structures (fascia, muscles, ligaments, tendons), leading to functional disturbances that increase the risk of back and peripheral joint pain or injuries [47]. Over time, these deviations may progress toward structural misalignments, underscoring the need for early detection. Despite the availability of advanced imaging techniques such as digital whole-body radiography, computed tomography, or magnetic resonance imaging, basic clinical examination remains highly valuable; in particular, the protocol and tool used in this study provide a rapid, reliable, and non-invasive method for identifying at-risk young people [20].

Significant sex-based differences were also found in hip ROM and spinal mobility. Girls outperformed boys in all ROMs, a finding consistent with previous studies [48,49,50], tests. These differences are likely explained by both anatomical (pelvic shape, spinal morphology) and hormonal influences (e.g., estrogen, relaxin) [51,52]. Notably, boys exhibited a higher prevalence of reduced hip extension (PHFKE test) and posterior pelvic tilt, suggesting a link between lower limb flexibility deficits—particularly in the hamstrings and hip flexors—and compensatory postural adaptations [2,11,12]. Given that reduced flexibility has been identified as a modifiable risk factor for LBP in children and adolescents [2], and is associated with altered lumbopelvic mechanics and increased spinal loading [53,54,55], these findings highlight clinically relevant targets for preventive programs, particularly in boys.

The last component of the “Postural Fitness” protocol assessed trunk muscle endurance. Boys demonstrated greater lateral flexor endurance (SB test), while girls performed better on the trunk extensor endurance test (BS test). These differences may reflect sex-related spinal morphology and muscle recruitment strategies and are consistent with earlier findings [56,57,58,59,60,61]. The enhanced extensor endurance in girls may reflect the mechanical advantage provided by their greater lumbar curvature, facilitating engagement of the erector spinae [45,62]. Boys’ superior lateral performance may relate to differences in muscle mass distribution and trunk geometry [63]. Importantly, endurance deficits in trunk endurance muscles have been linked to a poor postural control and increased risk of spinal pain [64,65], suggesting that both sexes could benefit from tailored trunk conditioning programs, but with different emphases.

Modifiable physical factors—such as flexibility and muscular endurance—influence spinal alignment and may contribute to BP symptoms if not addressed [3,10,16]. Identifying these deficits early is valuable for implementing preventive strategies in school or sports contexts. For example, children with greater thoracic kyphosis or reduced lumbar curvature, combined with reduced hip ROM and weak trunk endurance, have been shown to report higher pain intensity, poorer perceived health, and greater functional limitations [64,66]. Therefore, screening protocols that integrate postural evaluation with functional tests may improve early detection of at-risk individuals.

Understanding how these variables interact with growth, maturity, and sex can enhance the prevention and management of musculoskeletal conditions in youth. Preventive interventions focusing on physical, behavioral, and environmental factors can serve a dual purpose: reducing current discomfort and fostering healthier habits during critical developments. This aligns with evidence supporting the effectiveness of physiotherapy programs—including postural training, stretching, strengthening, and sensorimotor exercises—in reducing BP prevalence and improving postural health in children and adolescents [65,67]. School-based initiatives that combine ergonomic education, flexibility training, and core strengthening can be particularly effective. Functional and engaging modalities, such as corrective dance or movement games, have shown promising outcomes, especially for adolescent girls with thoracic hyperkyphosis [67]. Furthermore, psychosocial and lifestyle factors—such as screen time, backpack load, and quality of life—may also benefit from proper “Postural Fitness”. For instance, incorporating training programmes focused on health and postural awareness can help promote healthier sitting habits (e.g., during studying, watching TV, or using mobile devices) and reduce the discomfort associated with carrying heavy backpacks. This is particularly relevant given that most students exceed the recommended workplace limit for adults, generally set at 10% of body weight [55,68,69,70].

This study presents several limitations. First, its cross-sectional design prevents establishing causal relationships or assessing changes over time. A longitudinal approach would be necessary to determine the effect of age on postural alignment, particularly regarding variations in pelvic parameters and lumbar lordosis. Additionally, since the sample was drawn from a single region in Spain, the findings cannot be generalized to other regions or countries due to potential environmental, social, cultural, and genetic differences. Nonetheless, the results may still be valuable for developing preventive programs within the local community, particularly in educational settings. Furthermore, to properly understand the evolution of the variables assessed with the “Postural Fitness” protocol across childhood and adolescence, it is essential to account for participants’ developmental stage. Although this study included a large sample, the heterogeneous distribution of participants across sex- and stage-based subgroups prevented such an analysis. Future research should therefore stratify participants by developmental stage and sex from the beginning, ensuring adequate subgroup sizes to obtain robust and generalizable results. Finally, future research should incorporate BP records to examine their association with the variables assessed in the “Postural Fitness” protocol and to better determine the protocol’s clinical applicability and relevance.

## 5. Conclusions

In summary, this study highlights clear sex-related differences in “Postural Fitness” among children and adolescents. These findings emphasize the need for early, individualized, and context-specific interventions to optimize back health, reduce risk factors for BP, and promote lifelong musculoskeletal well-being.

Training programs for education professionals and parents can prepare them to correct students’ postural habits during daily activities. In addition, health education initiatives can be designed and implemented, focusing on the identification of risk factors, preventive strategies, and postural exercises for students. These interventions are particularly important because it is during the school years that children begin to establish habits and attitudes that will persist throughout their lives.

The “Postural Fitness” protocol offers a potentially useful framework for assessing back health and serves as a basis for developing individualized preventive strategies.

## Figures and Tables

**Table 1:** Reference values for the sagittal spinal assessment of thoracic and lumbar curve in each position [20].

Spinal Curve	SP		SSP		FTFP	
	Values	Classification	Values	Classification	Values	Classification
Thoracic	<20°	Hypokyphosis	<20°	Hypokyphosis	<40°	Hypokyphosis
	20° to 40°	Normal	20° to 40°	Normal	40° to 65°	Normal
	>40°	Hyperkyphosis	>40°	Hyperkyphosis	>65°	Hyperkyphosis
Lumbar	<−20°	Hypolordosis	<−15°	Lordosis	<10°	Hypokyphosis
	−20° to −40°	Normal	−15° to 15°	Normal	10° to 30°	Normal
	>40°	Hyperlordosis	>15°	Hyperkyphosis	>30°	Hyperkyphosis

FTFP = maximal forward trunk flexion position; SP = standing position; SSP = slump sitting position.

**Table 2:** Diagnostic classification of the SIM for the thoracic curve [20].

Classification	Subclassification	SP	SSP	FTFP
Normal kyphosis		Normal (20° to 40°)	Normal (20° to 40°)	Normal (40° to 65°)
Functional thoracic hyperkyphosis	Static	Normal (20° to 40°)	Hyperkyphosis (>40°)	Normal (40° to 65°)
	Dynamic	Normal (20° to 40°)	Normal (20° to 40°)	Hyperkyphosis (>65°)
	Total	Normal (20° to 40°)	Hyperkyphosis (>40°)	Hyperkyphosis (>65°)
Hyperkyphosis	Total	Hyperkyphosis (>40°)	Hyperkyphosis (>40°)	Hyperkyphosis (>65°)
	Standing	Hyperkyphosis (>40°)	Normal (20° to 40°)	Normal (40° to 65°)
	Static	Hyperkyphosis (>40°)	Hyperkyphosis (>40°)	Normal (40° to 65°)
	Dynamic	Hyperkyphosis (>40°)	Normal (20° to 40°)	Hyperkyphosis (>65°)
Hypokyphosis/Hypokyphotic attitude	Flat-back	Hypokyphosis (<20°)	Hypokyphosis (<20°)	Hypokyphosis (<40°)
	Standing	Hypokyphosis (<20°)	Normal (20° to 40°)	Normal (40° to 65°)
	Static	Hypokyphosis (<20°)	Hypokyphosis (<20°)	Normal (40° to 65°)
	Dynamic	Hypokyphosis (<20°)	Normal (20° to 40°)	Hypokyphosis (<40°)
Hypomobile kyphosis		Normal (20° to 40°)	Normal (20° to 40°)	Hypokyphosis (<40°)

FTFP = maximal forward trunk flexion position; SP = standing position; SSP = slump sitting position.

**Table 3:** Diagnostic classification of the SIM for the lumbar curve [20].

Classification	Subclassification	SP	SSP	FTFP
Normal lordosis		Normal (−20° to −40°)	Normal (−15° to 15°)	Normal (10° to 30°)
Lumbar spine with reduced mobility	Functional lumbar lordosis/Hypomobile lordosis	Normal (−20° to −40°)	Normal (−15° to 15°)	Hypokyphosis or lordosis (<10°)
	Lumbar hypomobility	Hypolordosis (<−20°)	Normal (−15° to 15°)	Hypokyphosis (<10°)
Hyperlordotic attitude		Hyperlordosis (>−40°)	Normal (−15° to 15°)	Normal (10° to 30°)
Functional lumbar hyperkyphosis	Static	Normal (−20° to −40°)	Hyperkyphosis (>15°)	Normal (10° to 30°)
	Dynamic	Normal (−20° to −40°)	Normal (−15° to 15°)	Hyperkyphosis (>30°)
	Total	Normal (−20° to −40°)	Hyperkyphosis (>15°)	Hyperkyphosis (>30°)
Lumbar hypermobility	Hypermobility 1	Hyperlordosis (>−40°)	Hyperkyphosis (>15°)	Hyperkyphosis (>30°)
	Hypermobility 2	Hyperlordosis (>−40°)	Normal (−15° to 15°)	Hyperkyphosis (> 30°)
	Hypermobility 3	Hyperlordosis (>−40°)	Hyperkyphosis (>15°)	Normal (10° to 30°)
Hypolordosis	Hypolordotic attitude	Hypolordosis (<−20°)	Normal (−15° to 15°)	Normal (10° to 30°)
	Lumbar kyphosis 1	Hypolordosis (<−20°)	Hyperkyphosis (>15°)	Hyperkyphosis (>30°)
	Lumbar kyphosis 2	Hypolordosis (<−20°)	Hyperkyphosis (> 15°)	Normal (10° to 30°)
	Lumbar kyphosis 3	Hypolordosis (<−20°)	Normal (−15° to 15°)	Hyperkyphosis (>30°)
Structured Hyperlordosis		Hyperlordosis (>−40°)	Hyperlordosis (<−15°) or normal (−15° to 15°)	Lordosis or Hypokyphosis (<10°)
Structured lumbar kyphosis		Hypolordosis or kyphosis (<−20°)	Hyperkyphosis (>15°)	Hyperkyphosis (>30°)

FTFP = maximal forward trunk flexion position; SP = standing position; SSP = slump sitting position.

**Table 4:** Descriptive statistics (mean ± SD) for anthropometric characteristics and “Fitness Postural” assessment in the total sample and by sex.

Variables	Total Sample (n = 494)	Male (n = 247)	Female (n = 247)
**Anthropometric Characteristics**			
Age (y)	11.03 ± 1.5 (10.9–11.2)	11 ± 1.5 (10.8–11.2)	11 ± 1.5 (10.8–11.2)
Body mass (kg)	44.3 ± 11.4 (43.2–45.3)	44 ± 10.9 (42.6–45.4)	44.5 ± 11.8 (43–46)
Body height (cm)	146.5 ± 9.7 (145.6–147.3)	146.9 ± 10.1 (145.7–148.2)	145.9 ± 9.3 (144.8–147.1)
BMI (kg/m^2^)	20.4 ± 3.8 (20.1–20.8)	20.2 ± 3.6 (19.7–20.6)	20.6 ± 4 (20.1–21.1)
**Fitness Postural Protocol**			
SIM			
SP thoracic (°)	38.5 ± 9.5 (37.7–39.4)	40.3 ± 9.6 (39.1–41.5)	36.7 ± 9.2 (35.5–37.8) *
SP lumbar (°)	33.6 ± 8.8 (32.8–34.5)	31.5 ± 8.5 (30.4–32.5)	35.7 ± 8.6 (34.6–36.8) *
SSP thoracic (°)	35.5 ± 10.3 (34.6–36.4)	37.2 ± 10.2 (35.9–38.5)	33.8 ± 10.2 (32.5–35.1) *
SSP lumbar (°)	11.1 ± 10.9 (10.1–12.1)	13.4 ± 11.4 (12–14.9)	8.7 ± 10 (7.5–10) *
FTFP thoracic (°)	49.3 ± 11.9 (48.3–50.4)	51.3 ± 11.1 (49.9–52.7)	47.4 ± 12.3 (45.9–48.9) *
FTFP lumbar (°)	26 ± 8.7 (25.2–26.8)	27.7 ± 8.4 (26.6–28.7)	24.3 ± 8.7 (23.2–25.4) *
Pelvic tilt and Toe Touch test			
L-H angle in SSP (°)	102.3 ± 8.6 (101.6–103.1)	104.9 ± 8.4 (103.8–105.9)	99.7 ± 8.1 (98.6–100.7) *
L-V angle in FTFP (°)	114.5 ± 14.9 (113.1–115.9)	120.6 ± 12 (119–122.2)	108.3 ± 15 (106.2–110.3) *
TT test (cm)	−7.3 ± 8.6 (−8.1–(−6.6))	−10.2 ± 7.5 (−11.1-(−9.3))	−4.5 ± 8.7 (−5.6–(−3.4)) *
ROM			
PHE-R (°)	17.7 ± 8.6 (16.9–18.4)	16.8 ± 8.1 (15.8–17.8)	18.5 ± 9 (17.4–19.7) *
PHE-L (°)	17.6 ± 8.2 (16.9–18.4)	16.8 ± 7.6 (15.9–17.8)	18.5 ± 8.7 (17.4–19.6) *
PHFKE-R (°)	74.6 ± 29 (72–77.2)	68.9 ± 8.6 (67.4–69.6)	77.9 ± 13 (75.5–78.7) *
PHFKE-L (°)	72.8 ± 11.8 (71.7–73.8)	68.5 ± 8.9 (67.9–70.1)	77.1 ± 12.7 (75.3–85.1) *
Trunk Muscle Endurance			
Ito test (s)	95.8 ± 72 (85.9–105.6)	106.9 ± 82.1 (89.6–124.2)	84.7 ± 61.9 (75.4–94)
BS test (s)	114.7 ± 72.7 (107.9–121.4)	106.2 ± 69.8 (97–115.4)	123.2 ± 74.7 (113.3–133.1) *
SB-R test (s)	45 ± 28.3 (42.5–47.6)	48.7 ± 31 (44.7–52.6)	41.4 ± 24.9 (38.2–44.5) *
SB-L test (s)	45.9 ± 29.3 (43.3–48.6)	50.9 ± 32.5 (46.8–55.1)	40.9 ± 24.7 (37.8–44.1) *

* Significant differences between boys and girls (*p* < 0.05). BMI = body mass index; BS = Biering–Sørensen; cm = centimeters; FTFP = maximal forward trunk flexion position; kg = kilograms; L-H = lumbo-horizontal; L-V = lumbo-vertical; PHE (R-L) = passive hip extension (right-left); PHFKE (R-L) = passive hip flexion with the knee extended (right-left); ROM = range of motion; s = seconds; SB (R-L) = Side Bridge (right-left); SIM = Sagittal Integral Morphotype; SP = standing position; SSP = slump sitting position; TT = Toe Touch test; y = years; ° = degrees.

**Table 5:** Distribution of participants (%) and counts by spinal curve according to assessment position and classification by SIM; presented for the total sample and stratified by sex.

	Classification	Total Sample (n = 494)	Male (n = 247)	Female (n = 247)	Chi-Square
**Thoracic curvature**					
SP	Hypokyphosis	0.8% (4)	0% (0)	1.6% (4)	χ^2^_(2)_ = 9.45, *p* = 0.01
	Normal	58.2% (289)	53.8% (133)	63.1% (156)	
	Hyperkyphosis	40.6% (201)	46.1% (114)	35.2% (87)	
SSP	Hypokyphosis	1.7% (4)	0% (0)	1.7% (4)	χ^2^_(2)_ = 8.43, *p* = 0.01
	Normal	71.2% (352)	67.6% (167)	74.9% (185)	
	Hyperkyphosis	27.9% (138)	32.4% (80)	23.5% (58)	
FTFP	Hypokyphosis	0.4% (1)	0% (0)	0.4% (1)	χ^2^_(2)_ = 7.577, *p* = 0.02
	Normal	26.9% (133)	21.8% (54)	31.9% (79)	
	Hyperkyphosis	72.9% (360)	78.2% (193)	67.6% (167)	
SIM	Normal kyphosis	16.2% (80)	12.1% (30)	20.2% (50)	χ^2^_(9)_ = 21.774, *p* = 0.01
	Functional static hyperkyphosis	2% (10)	1.2% (3)	2.8% (7)	
	Functional dynamic hyperkyphosis	32% (158)	30.8% (76)	33.2% (82)	
	Total functional hyperkyphosis	8.1% (40)	9.7% (24)	6.5% (16)	
	Hyperkyphosis standing	6.3% (31)	5.7% (14)	6.9% (17)	
	Hyperkyphosis static	1.6% (8)	2.8% (7)	0.4% (1)	
	Hyperkyphosis dynamic	16.6% (82)	19% (47)	174.2% (35)	
	Total hyperkyphosis	16.2% (80)	18.6% (46)	13.8% (34)	
	Hypokyphosis	0.8% (4)	0% (0)	1.6% (4)	
	Hypomobile kyphosis	0.2% (1)	0% (0)	0.4% (1)	
**Lumbar curvature**					
SP	Hypolordosis	4.8% (24)	6.47% (16)	3.2% (8)	χ^2^_(2)_ = 11.989, *p* = 0.002
	Normal	77.5% (383)	81.4% (201)	73.7% (182)	
	Hyperlordosis	17.6% (87)	12.1% (30)	23.1% (57)	
SSP	Lordosis	0% (0)	0% (0)	0% (0)	χ^2^_(1)_ = 17.386, *p* < 0.001
	Normal	64.3% (317)	55.3% (136)	73.3% (181)	
	Hyperkyphosis	35.7% (176)	44.7% (110)	26.7% (66)	
FTFP	Hypokyphosis	2.2% (11)	1.6% (4)	2.8% (7)	χ^2^_(2)_ = 3.64, *p* = 0.16
	Normal	72.1% (356)	69.2% (171)	74.9% (185)	
	Hyperkyphosis	25.7% (127)	29.1% (72)	22.2% (55)	
SIM	Normal lordosis	38.9% (192)	35.2% (87)	42.5% (105)	χ^2^_(10)_ = 25.536, *p* = 0.004
	Functional lumbar lordosis	1.6% (8)	1.2% (3)	2% (5)	
	Hyperlordotic attitude	14.4% (71)	9.7% (24)	19% (47)	
	Functional static hyperkyphosis	15% (74)	19.8% (49)	10.1% (25)	
	Functional dynamic hyperkyphosis	7.1% (35)	6.9% (17)	7.3% (18)	
	Total Functional hyperkyphosis	15% (74)	18.2% (45)	11.7% (29)	
	Lumbar hypermobility	2.6% (13)	2% (5)	3.2% (8)	
	Hypolordotic attitude	1.2% (6)	1.2% (3)	1.2% (3)	
	Lumbar kyphosis	2% (10)	2.8% (7)	1.2% (3)	
	Structured hyperlordosis	0.6% (3)	0.4% (1)	0.8% (2)	
	Structured lumbar kyphosis	1.6% (8)	2.4% (6)	0.8% (2)	

FTFP = maximal forward trunk flexion position; SIM = Sagittal Integral Morphotype; SP = standing position; SSP = slump sitting position.

**Table 6:** Distribution of participants (%) and counts by pelvic tilt and ROM; presented for the total sample and stratified by sex.

	Classification	Total Sample (n = 494)	Male (n = 247)	Female (n = 247)	Chi-Square
**Pelvic tilt**					
L-H angle	Neutral	43.9% (212)	31.7% (78)	56.5% (134)	χ^2^_(1)_ = 30.226, *p* < 0.001
	Posterior PT	56.1% (271)	68.3% (168)	43.4% (103)	
L-V angle	Neutral	20.1% (85)	5.6% (12)	35.1% (73)	χ^2^_(1)_ = 57.69, *p* < 0.001
	Posterior PT	79.9% (339)	94.4% (204)	64.9% (135)	
**ROM**					
PHE-R	Normal	70.6% (349)	67.2% (166)	74.1% (183)	χ^2^_(1)_ = 2.82, *p* = 0.08
	Reduced	29.4% (145)	32.8% (81)	25.9% (64)	
PHE-L	Normal	71.6% (354)	69.2% (171)	74.1% (183)	χ^2^_(1)_ = 1.43, *p* = 0.23
	Reduced	28.4% (140)	30.8% (76)	25.9% (64)	
PHFKE-R	Normal	37.9% (187)	22.7% (56)	53.1% (131)	χ^2^_(1)_ = 48.403, *p* < 0.001
	Reduced	62.1% (307)	77.3% (191)	46.9% (116)	
PHFKE-L	Normal	38.9% (192)	25.1% (62)	52.6% (130)	χ^2^_(1)_ = 39.39, *p* = 0.001
	Reduced	61.1% (302)	74.9% (185)	47.4% (117)	

L-H = lumbo-horizontal; L-V = lumbo-vertical; PHE (R-L) = passive hip extension (right/left); PHFKE (R-L) = passive hip flexion with the knee extended (right/left); ROM = range of motion; PT: pelvic tilt.

## Data Availability

The data presented in this study are available on request from the corresponding author. The data are not publicly available due to privacy.

## Abbreviations

The following abbreviations are used in this manuscript:

BP	Back pain
BMI	Body mass index
ROM	Range of motion
PE	Physical education
SIM	Sagittal Integral Morphotype
SP	Standing position
SSP	Slump sitting position
FTFP	Forward trunk flexion position
TT	Toe Touch test
L-H	Lumbo-horizontal angle
L-V	Lumbo-vertical angle
PHFKE	Passive hip flexion with knee extended
PHE	Passive hip extension
BS	Biering–Sørensen test
SB	Side Bridge test
